# Basis for the diversity and extent in loss of digestible nutrients created by dietary phytin: Emphasis on fowl and swine

**DOI:** 10.1016/j.aninu.2023.11.010

**Published:** 2024-01-08

**Authors:** Edwin T. Moran, Michael R. Bedford

**Affiliations:** a Poultry Science Department, Auburn University, AL, 36830-5416, USA; b AB Vista, Woodstock Court, Blenheim Road, Marlborough, Wiltshire, SN8 4AN, UK

**Keywords:** Digestive enzyme, Calcium, Endogenous protein, Mucin, Phytin

## Abstract

Phytin is the Ca^2+^-Mg^2+^-K^+^ salt of phytic acid that is created and deposited in the aleurone layer and/or germ of grains and legumes. Its high presence in feedstuffs for fowl and swine diets results in it being a universal and significant impediment to optimum performance. Phytin impairs gastrointestinal recovery of a wide array of nutrients, the effect varying with the nutrient concerned. On exposure to low pH during gastric digestion, phytin dissociates into phytic acid and solubilized Ca^2+^. Even at low gastric pH, phytic acid is negatively charged which forms the basis of its anti-nutritive behavior. Pepsinogen has extensive basic amino acids on its activation peptide that are presented as cations at low pH which are targeted by pepsin for activation. Partially crystalized Ca^2+^ near the enzyme's active site further stabilizes its newly formed structure. Thus, phytic acid appears to interfere with gastric digestion by several mechanisms; interfering with pepsinogen activation by binding to the polypeptide's basic amino acids; coordinating free Ca^2+^, destabilizing pepsin; binding some dietary proteins directly, further compromising gastric proteolysis. Upon digesta attaining neutrality in the duodenum, Ca^2+^ and other cations re-bind with accessible anions, phytic acid being a significant contender. Phytate not only binds free cations but can also strip them from enzymes (e.g. Ca^2+^, Zn^2+^) which reduces their structural resistance to autolysis and ability as co-factors (e.g. Zn^2+^) to increase enzyme activity. Goblet cells initially employ Ca^2+^ as an electronic shield between mucin layers enabling granule formation and cell storage. After mucin granule release, Ca^2+^ is progressively displaced by Na^+^ to free the viscous mucins enabling its translocation. Mucin entangles with the glycocalyx of adjacent enterocytes thereby constructing the unstirred water layer (USWL). Excessive removal of Ca^2+^ from mucin by phytic acid increases its fluidity facilitating its loss from the USWL with its associated Na^+^. This partly explains increased mucin and Na^+^ losses noted with high phytate diets. This review suggests that phytic acid binding of Ca^2+^ and less so Zn^2+^ is the basis for the diversity in nutrient losses encountered and that such losses are in proportion to dietary phytate content.

## 1 Introduction

Phytin refers to the Ca^2+^-Mg^2+^-K^+^ salt of phytic acid that is primarily formed within the aleurone layer and/or germ of the grains and legumes (Ravindran et al., 1994). An overwhelming use of these feedstuffs in commercial rations provides considerable phytin for fowl and swine upon consumption. Unfortunately, the associated phosphorus is not only indigestible, but dietary phytin interferes with the recovery of a wide array of nutrients (Cowieson et al., 2008, Cowieson et al., 2004; Selle et al., 2009; Woyengo et al., 2009). As a result, a proportion of dietary energy as either starch or fat, as well as protein regardless of amino acid composition, and many minerals are not realized.

**Fig. 1:**
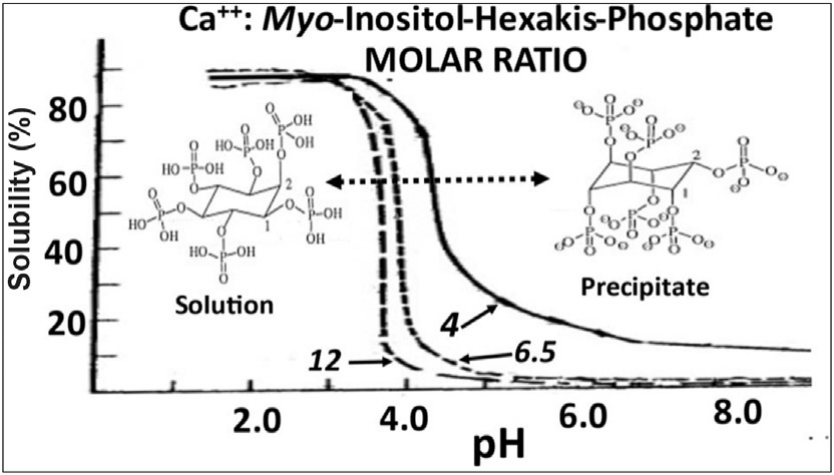
The relationship between Ca^2+^ to *myo*-inositol-hexa-phosphate molar ratio and the solubility of Ca^2+^ with changing pH. Modification of selected graphics (Grynspan and Cheryan, 1983; Martin and Evans, 1986; Veiga et al., 2014) were used to assemble the present figure.

Binding by *myo-*inositol hexaphosphate predominates with the alkali and first transition series elements. While association with the respective mono-valents continues phytic acid solubility, binding by the di-valents creates a complex that eventually precipitates as pH approaches neutrality. Differences in binding affinities for phytic acid exist among the di-valents because of variation in size and charge, the latter being determined by the pH of the solution. Vohra et al. (1965) reported that the binding affinity of Cu^2+^ > Zn^2+^ > Ni^2+^ > Co^2+^ > Mn^2+^ > Fe^2+^ > Ca^2+^ when 5 mol of the divalent ion was added to 1 mol of sodium phytate at pH 7.4. Such differences in binding have to be viewed in context however, because in practical terms their relative concentrations in typical monogastric diets vary markedly from that investigated in vitro (Ca^2+^ being present in far greater concentrations than Cu^2+^ for example). Furthermore, the environmental pH markedly influences the concentration of di-valent cation required to initiate precipitation as exemplified by Ca^2+^ (Fig. 1). Phytin complexes proceed from dissociated soluble ionic forms under acid terms to those being simple soluble complexes near neutrality that eventually precipitate when alkaline (Grynspan and Cheryan, 1983; Martin and Evans, 1986; Marolt et al., 2020). The transition of soluble to insoluble calcium-phytate is due to a change in phytate conformation from five equatorial and one axial phosphate groups to five axial and one equatorial group (Fig. 1). The phosphate residing at each of the inositol carbons differs in its ionization potential and associated p*Ka* when in solution. In this respect, the phosphates at C-2 and C-5 would seem to be particularly prone to binding cations during digesta passage into the small intestine as a result of the p*Ka* of their transition from mono-to di-anion occurring close to neutrality (Table 1).

**Table 1:** Carbon location on the inositol ring and p*Ka*^1^ potential for binding.

Inositol carbon #	Transition p*Ka*	
	None to mono-anionic	Mono- to di-anionic
1	2.1	12.0
2	1.1	6.8
3	1.5	12.0
4	1.7	10.0
5	1.7	7.6
6	1.7	10.0

Approximation values obtained by nuclear magnetic resonance pH titration (Costello et al., 1976).

1 p*Ka* is the negative base-10 logarithm of the acid dissociation constant (*Ka*) of a solution.

Phytase supplementation has been clearly shown to improve the availability of phytate bound phosphorus together with many other nutrients (Kies et al., 2001; Selle et al., 2006; Pirgozliev et al., 2012; Zaefarian et al., 2013). Successive release of ring phosphates by super dosing with phytase results in production of progressively lower esters which are proportionately less able to chelate cations (Graf, 1983; Kaufman and Kleinberg, 1971; Persson et al., 1998; Liau et al., 2005). The specific complexing of phytic acid with Ca^2+^ can be expected to dominate within the small intestine because of readily accessible quantities at-hand. Selle et al. (2009) commented that phytate may bind and thus remove approximately one third of dietary Ca^2+^. Whilst dietary Ca^2+^ undoubtedly is the source of the majority of intestinal Ca^2+^, two other lesser-known sources should be considered, namely the pancreatic enzymes and the mucin forming the unstirred water layer (USWL). The point to be made here is that phytic acid is capable of stripping the Ca^2+^ out of these endogenous proteins and in doing so severely compromise their functionality.

The following rationalizes that Ca^2+^ binding is the basis for a significant proportion of the variability in quantity of nutrients lost because of dietary phytin. This expands on the current tenet that direct binding of dietary proteins or pepsinogen by phytin explains the majority of lost nutrients observed (Selle and Ravindran, 2007, 2008; Selle et al., 2012). Such binding to Ca^2+^ not only reduces the effectiveness of gastric and pancreatic enzymes but also facilitates mucin release from the lumen surface. Addressing both topics necessitates a broad array of references to support this proposal which we admit is speculation in some part. Reviews have been referenced to condense thought with introductory aspects whereas research papers are preferred when direct implications are involved.

## 2 Ca^2+^ and digestion

Pepsin is the first enzyme encountered by ingesta that has a direct association with Ca^2+^ and consequently suffers from exposure to phytin. Camus and Laporte (1976) demonstrated that the in vitro proteolysis of casein using pepsin could be inhibited by phytin derived from wheat by-products as well as in pure form. The formation of insoluble protein chelates at low pH with phytic acid was offered as the basis for this inhibition. Liu et al., 2009, Liu et al., 2010 found that the pepsin activity from proventricular contents from broilers given dietary phytin was reduced but then increased when phytase was supplemented. The question to consider is whether phytate is interfering with pepsin or the substrate or perhaps both.

The amino acid compositions of pepsin and pepsinogen show considerable homology between species (Baudyŝ and Kostka, 1983; Ong and Perlmann, 1968). Many isoenzymes of pepsinogen exist which are all activated by a reduction in pH (<5) that initiates autolysis of its amino end polypeptide (Stanforth et al., 2022). The activation peptide has extensive sequences of basic amino acids that presumably alter the enzyme structure upon cation formation at low pH to effect cleavage (Ong and Perlmann, 1968). Yang et al. (2003) observed a subsequent crystallization with Ca^2+^ once pepsin was formed that appeared to stabilize the active site. The high concentration of cationic amino acids on the activation peptide at gastric pH provide an opportunity for binding with phytic acid's anionic groups which is envisaged as the basis for impairing pepsinogen's activation. Similarly, dietary proteins which carry significant cationic sequences likely bind with phytic acid, further interfering with pepsin proteolysis. Finally, the Ca^2+^ ions responsible for stabilizing the newly formed pepsin molecule are subject to extraction by phytate, further challenging gastric proteolysis.

The low pH of the gastric environment places charges on basic amino acids and removes charges from acidic groups of the dietary proteins, ultimately introducing a significant strain on the three-dimensional structure of the protein, exposing epitopes to pepsin digestion that would not be available at a higher pH. Gastric digestion focuses pepsin proteolysis at clusters of hydrophobic amino acids in the dietary protein that concentrate around cystine disulfide bonds which link peptide chains having a parallel arrangement. Their cleavage improves protein compatibility with water while the low pH separately coalesces the array of dietary lipids (Moran, 2016), ultimately improving protein digestion by reducing interactions between fats and hydrophobic proteins. Upon entry into the duodenum the gastric milieu is buffered to approach neutrality, straining the structures of the dietary protein once more. The pancreatic enzymes immediately enter the duodenal lumen in swine whereas fowl retro-return them from the jejunal juncture (Moran, 1982). Many subtle differences exist in the physiological approach to effect digestion between fowl and swine through the small intestine, but the overall results are very similar (Moran, 2022).

Small intestinal proteolysis proceeds through trypsin and chymotrypsin to yield large polypeptides that are further reduced by carboxypeptidases A (aromatic or branch chain targets) and B (basic amino acids). These exo-acting enzymes favour hydrolysis at sequences where essential amino acids tend to be present. Consequently, the resulting end products are largely the essential amino acids in free form that intestinal motility conveys through the USWL with accompanying peptides being dominated by the nonessential amino acids that encounter further reduction at the mucosa before absorption (Moran, 2019). Trypsin is particularly important in all small intestinal proteolysis because of its activation of all proenzymes from the pancreas including itself. Thus, the recognition that phytin can inhibit trypsin both in vitro and in vivo, an effect which can be relieved by co-administration of dietary phytase (Singh and Krikorian, 1982; Liu et al., 2009, Liu et al., 2010) highlights the marked effects this molecule can have on small intestinal proteolysis.

Trypsin within the lumen can activate newly released trypsinogen as can sloughed mucosal peptidases which act as an alternative (Kotormán et al., 2003). Trypsinogen has two binding sites for Ca^2+^ with the weak one being lost once it is activated while the tight one remains to enhance structure thereby minimizing threat from other proteolytic enzymes. Caldwell (1992) related that complexation of the loose Ca^2+^ by phytin increases the rate of trypsinogen activation approximately 30-fold whereas removal of the tight one decreases trypsin's duration of proteolytic effectiveness 100-fold. Either of these activities will clearly compromise proteolysis by excessive commitment of the zymogen when it is not needed, and by reduction in the overall proteolytic capacity. Chymotrypsin catalyses the degradation of trypsin by cleavage of the dependent Ca^2+^ binding loop far more effectively when the Ca^2+^ is removed (Wu and Laskowski, 1956; Szabó and Sahin-Tóth, 2012). A further antinutritive aspect of Ca^2+^ binding is evident in this regard because this cleaving activity of chymotrypsin is suppressed when chymotrypsin itself is bound by Ca^2+^.

Carboxypeptidase A and B degrade large peptides resulting from trypsin and chymotrypsin digestion with both exopeptidases depending on Zn^2+^ at their active site. The resultant amino acids and small peptides may subsequently pass through the USWL. Peptidase activity that exists below the USWL also depends on Zn^2+^ to finalize proteolysis for absorption while also activating trypsinogen as enterocytes are sloughed into the lumen. Although phytin has been shown to reduce activity of these peptidases, any implication of Ca^2+^ is lacking compared to Zn^2+^ (Liu et al., 2009, Liu et al., 2010; Yu et al., 2010), and thus stripping of Zn^2+^ from the active sites of these enzymes by phytate is most likely the mechanism at play in this situation. Advantages in live performance from phytase supplementation have been shown to occur with fowl and swine when their diet is limiting in Zn^2+^ (Jondreville et al., 2007; Schlegel et al., 2010). Given that Zn^2+^ has a greater relative binding capacity than Ca^2+^ (Vohra and Kratzer, 1965) for phytate a preferential response to phytase can be expected even though its concentration would be low in comparison with Ca^2+^.

α-Amylase focuses on starch and its degradation to maltose, maltotriose and α-limit dextrin which are further reduced to glucose at the enterocyte surface for absorption. The α-amylases of fowl and swine exhibit very similar characteristics, and both are highly dependent on Ca^2+^ for activity (Buonocore et al., 1977; Buisson et al., 1987). Most sources of α-amylase also have a similar homology that suffers in activity upon complexing of their Ca^2+^ by powerful chelating agents (Chauncey et al., 1963). Stability is also compromised if Ca^2+^ is removed. Igarashi et al. (1998) demonstrated a greatly improved thermostability due to complexing of Ca^2+^ by a *Bacillus* α-amylase. Similarly, Tanaka and Hoshino (2002) observed that Ca^2+^ binding by the α-amylase of *Bacillus amyloliquifaciens* resulted in a reduction in its subsequent rate of inactivation. Saboury (2002), using the same organism demonstrated an improved stability and activity of α-amylase due to its binding of Ca^2+^. Phytic acid depresses the in vivo digestion of starch by α-amylase which can be relieved by phytase inclusion (Deshpande and Cheryan, 1984; Knuckles and Betschart, 1987) suggesting a reduction in starch digestibility may be mediated in part through Ca^2+^ removal. The lesser inositol phosphate esters were not as effective at the binding of Ca^2+^ and depression of amylase activity and hence removal of the *myo*-inositol hexakisphosphate (InsP6) is probably all that is required to affect an improvement in Ca^2+^ availability. In contrast, the chelation of Zn^2+^ is significant by InsP6, InsP5, InsP4 and even InsP3, particularly at neutral pH, suggesting far more extensive phytate hydrolysis is required to enable full activation of Zn^2+^ dependent enzymes.

Fulfilling the digestion of dietary fat like that for protein requires several pancreatic enzymes to be present and active in the lumen. Lipase, co-lipase, and phospholipase A_2_ require activation of their respective pro-enzymes by trypsin with their collective efforts being performed on the fat droplet surface (Moran, 2021). These proteins are hydrophilic and need to be resistant to denaturation at the fat droplet's hydrophobic surface. Lipase together with co-lipase locate on top of the bile acid emulsifier during digestion thereby avoiding direct contact with the fat droplet. Access to Ca^2+^ facilitates lipase's continuance of in vitro activity and protection from denaturation (Alvarez and Stella, 1989) whereas inclusion of calcium-binding agents is inhibitory and decreases the rate of fat digestion (Hu et al., 2010). Indeed, lumenal lipase activity has been observed to decrease more rapidly in high compared with low phytate diets, suggesting lipase autolysis is accelerated if Ca^2+^ is limiting as it proceeds along the small intestine (Table 2).

**Table 2:** Reduction in the mucosal lipase activity with increasing phytate content (μmol/g protein).^1^

Item	Dietary phytin		*P-*value
	0.20%	0.40%	
Duodenum	1.15	1.04	<0.05
Jejunum	0.90	0.82	<0.05
Ileum	0.37	0.34	<0.05

1 Broiler chickens at 42 days of age given corn-soybean meal feed with added sodium phytate. Selected data from Liu et al., 2009, Liu et al., 2010.

Phospholipase A_2_, being an amphiphile, permits its direct association with the droplet surface where the hydrolysis of the 2-acyl fatty acids from all phospholipids occurs. Once again, Ca^2+^ is included at two sites within this enzyme that provides protection from denaturation while also delaying autolysis by trypsin (Pieterson et al., 1974; Drakenberg et al., 1984). In this regard, dietary phytin has been shown to interfere with fat digestion while phytase supplementation improved the recovery of all associated fatty acids equivalently (Liu et al., 2009, Liu et al., 2010; Zaefarian et al., 2013), implicating Ca chelation in this anti-nutritive effect.

## 3 Phytin and lumen mucosa

The integrity of the lumen mucosa is heavily dependent on the mutual functioning of the goblet cells with the enterocytes on each villus. Enterocytes could not perform their digestion-absorption activities without the mucin layer provided by the goblet cells. Both types of cells arise from the crypt and develop their respective talents during progression to the villus apex (Duangnumsawang et al., 2021). Both types of cells depend upon one another and are presented in a mosaic pattern on the villus surface. The enterocytes benefit by receiving a mucin covering of their surface just as goblet cells do through access to absorbed nutrients for the reciprocal support of mucin synthesis.

As goblet cells progress up the villus structure, mucin is synthesized for assembly into granules which accrue and are released as needed (Bansil and Turner, 2018). Soluble mucin (mucin-2 [MUC2]) employs significant amounts of threonine and serine as side chains on the core protein and the rate of production is commensurate with protein synthesis at the base of each cell. The subsequent passage of the mucin though the adjacent Golgi apparatus results in the formation of O-links of the associated oligosaccharides to the aforementioned hydroxy amino acids that surround and protect the core protein from proteolysis. These protein-oligosaccharide units are interconnected in a manner that creates macromolecules resembling a “fishnet.” Sialic acid content has been used to define the total amount of mucin. Other sugar members are also used as they are unique by defying hydrolysis while contributing carboxylate and/or sulfated groups that micro-buffer the pH of the associated USWL. Continual formation of these macromolecules necessitates their condensation into granule form for cell storage. The layering of these molecules requires extensive Ca^2+^ incorporation into this structure which acts as an electronic shield between the charged oligosaccharides, enabling significant reduction in mucin granule size (Paz et al., 2003). Clearly a reduction in Ca availability will compromise this process, again highlighting the potential for interference by phytin.

Expression of sulfate or carboxyl groups onto the component oligosaccharides leads to either acid or neutral staining of the mucins and therefore of the goblet cells that produce them. The acidic and neutral goblet cells are differentially expressed on the villi in line with the needs of the intestine. Both types of cell are found in the small intestine of fowl and swine (Pastor et al., 1988; Moré et al., 1987). Their proportions can vary from duodenum to ileum as well as with diet composition. The release of mucin granules from storage would seem to be equivalent between neutral and acid staining cells on the villi, and when these two types of mucin are secreted they buffer surface pH accordingly. The mucin granules once released progressively imbibe water and swell, creating viscous mucins with the presence of Ca^2+^ being essential to this viscous structure. Over time the Ca^2+^ is progressively removed and replaced by Na^+^ (Ambort et al., 2012; Sharma et al., 2020). Indeed, the in vitro studies of Curnutt et al. (2020) with mucin solutions demonstrated that the presence of Ca^2+^ decreases the transition of mucins from a gel to a soluble form, the soluble form presumably being more prone to escape the glycocalyx and loose mucin layer and thus be lost to the digesta.

The villi present a mosaic of enterocyte and goblet cells that are continuously receiving mucin or MUC2 that covers the surface. In essence, the enterocyte's glycocalyx captures the MUC2 as it is secreted during motility and it adheres to combine and form the USWL. MUC2's associated “fishnet” like structure acts as a molecular sieve to protect surface enzymes from both the pancreatic enzymes and microflora while limiting access to their products (Moran, 1982). MUC2's associated oligosaccharides not only protect the mucins underlying protein core but act as a localized buffer to optimize surface pH terms to finalize digestion and nutrient absorption (Shiau et al., 1985).

**Fig. 2:**
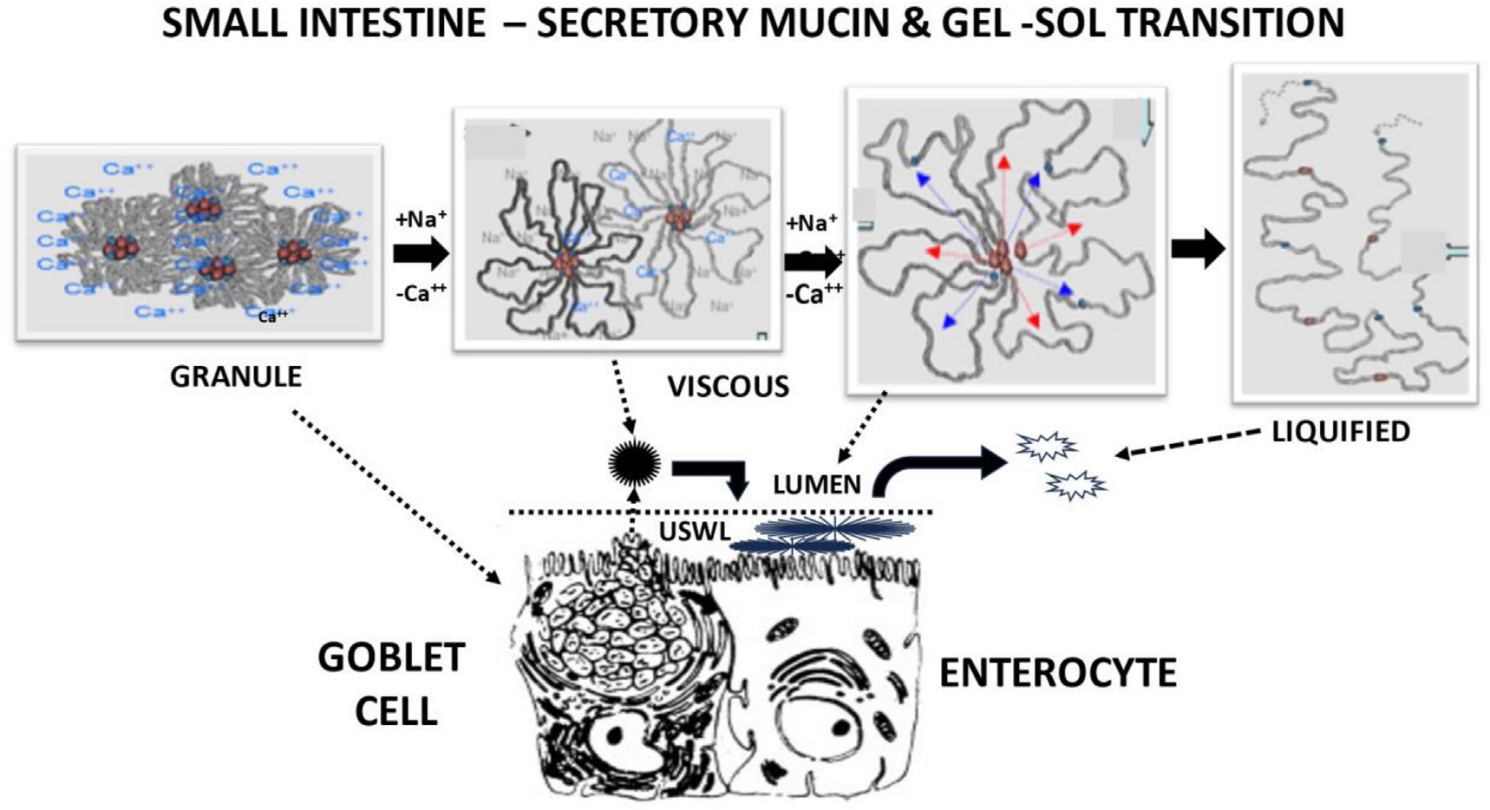
Expected modifications with secretory mucin produced by goblet cells of the small intestine and turnover at the unstirred water layer. Fig. 2 is a composite of portions redrawn from multiple illustrations (Freeman and Geer, 1965; Moran, 1982; Kesimer et al., 2010). Kesimer et al. (2010) provided the concept of Ca^2+^ substitution by Na^+^ with mucin-5B (MUC5B) as the means to liquify this mucin by the lungs. A parallel situation is speculated to occur with mucin-2 (MUC2) in the small intestine.

The integrity of the USWL at the lumen surface is subject to weaking of its gel structure. Sellers et al. (1991) demonstrated that the rheology of the pig's small intestinal mucus could be disrupted by an acid pH, detergents such as bile, and protein denaturants. Kesimer et al. (2010) rationalized that granular MUC5B associated with the lungs progresses through viscosity to liquidity by an orderly displacement of Ca^2+^ with Na^+^. Akter et al. (2019) reported that high dietary Na^+^ with broilers reduced retention of Ca^2+^, Mg^2+^ and phosphorus while supplementary phytase improved their digestibility as well as improving AME. The implication is that an aggressive binding of Ca^2+^ by phytic acid could accentuate liquification of mucin at the USWL surface thereby facilitating its lumen entry to increase endogenous loss (Fig. 2) especially as lumenal pH increases through the small intestine and thus the Ca^2+^ binding capacity of phytate increases (Table 3). Onyango et al. (2009) noted that the increased endogenous loss of broilers by dietary phytin could be largely attributed to mucin, supportive of this proposed theory. In summary, Ca^2+^ plays a vital role in mucin storage and functionality once secreted, and we propose that interference with Ca^2+^ availability by phytin is a principal cause of increased endogenous losses noted when high phytate diets are fed. As a consequence, the benefits noted when significant amounts of phytase are fed are likely due to a marked increase in availability of di-valent cations which play a crucial role in the digestive process and intestinal stability.

**Table 3:** Total micromole Ca^2+^ bound by one micromole phytic acid as altered by pH.^1^

Solution pH	Solutions with increased molar ratios of Ca^2+^ to phytic acid				
	1	2	4	6	8
4.4	0.00	0.00	0.00	0.00	0.00
4.8	0.00	0.00	0.00	0.00	0.10
5.2	0.00	0.10	0.20	0.00	0.40
5.6	0.16	0.20	0.80	1.30	1.70
6.0	0.34	0.40	1.90	3.10	2.80
6.4	0.47	0.90	2.70	3.85	3.60
6.8	0.53	1.51	3.46	4.10	3.90
7.2	0.71	1.67	3.63	4.28	4.00

1 Selected data from Martin and Evans (1986).

## 4 Overview

Phytin is located in many plant source feedstuffs, particularly those consumed by fowl and swine. Once consumed, commercially meaningful losses in digestibility of various nutrients occurs. Use of supplemental phytase can be broadly translated into a live production advantage but only witnessed when those nutrients are limiting to requirement. The fundamental basis for these losses and differences in extent can largely be attributed to the binding of Ca^2+^ by phytic acid within the gastro-intestinal tract (GIT). Dietary derived Ca^2+^ has to date been the focus of this chelating effect; however, substantial Ca^2+^ can be removed from gastric and pancreatic enzymes as well as from mucin.

Activation and stability of the gastric and pancreatic enzymes while in the lumen performing digestion can be impaired by deletion of Ca^2+^ from their structure. Such structural inclusion extends their digestive effectiveness that would otherwise rapidly be collectively lost. Essentially, nutrients within readily digestible feedstuffs would continue to be well recovered early in the small intestine, however the more slowly digested material would move distally as the digestive enzymes dissipate and as a result nutrients are lost to the large intestine, with potentially damaging effects. Connective tissue proteins, resistant starch and fats having long chain saturated fatty acids would experience reduced recovery before reaching the ileum.

Delaying pancreatic enzyme autolysis would not only enhance recovery of difficult nutrient sources but also complement the nutrient needs of the distal mucosa. Soufrant et al. (1993) notes that dietary casein is completely recovered by pigs before the ileum, whereas salvage of endogenous nutrients approximates 79%. Poorly digestible proteins typically provide inadequate amounts of limiting amino acids distally, whereas a concurrent release of endogenous sources can act to improve balance favorable for villi maintenance in the lower intestinal tract.

Mucin produced by goblet cells on the villus is released to entangle with the glycocalyx on microvilli of adjacent enterocytes. Mucin formation initially depends on Ca^2+^ in the assembly of granules. When these granules are released, they are exposed to accessible Na^+^ that displaces Ca^2+^ which over time decreases mucin's thixotropic character and ability to associated with the glycocalyx. With such changes the mucin is ultimately lost to the digesta and if unchecked it would continually reduce the USWL thickness if replacement were not adequate. Replacement mucins are expected to undergo continual modification of structure based on the nutrient and microbial conditions of the lumen. However, phytin presence would seem to over emphasize Ca^2+^ removal accentuating the rate of viscosity loss of the mucins and their ultimate endogenous loss. Fortunately, this filamentous mucin released from the ileum may be of subsequent advantage to the large intestine by re-enforcing the USWL's loose second layer from microbiological threat (Moran and Bedford, 2023), but small intestinal losses may become excessive.

Nutrient losses attributed to phytin discussed so far relate to activities within the GIT; however, it is possible that such losses can occur prior to feed consumption. Dendougui and Schwedt (2004) treated human foods in vitro under simulated gastric terms while measuring the proportion of Ca^2+^ bound by phytic acid. There were significant differences between the ingredients investigated. For example, Ca^2+^ was observed to be more extensively bound by soybean, chickpea, and oat samples compared with rice or corn semolina. Separately, Bressanti et al. (2002) boiled whole corn with lime water as if making tortillas (nixtamalization) then examined the Ca^2+^ and phytic acid contents in different parts of the grain. The bound Ca^2+^ concentration increased 18 times in whole corn and endosperm after cooking while germ escalated 24 times, whereas free phytic acid per se decreased 27.9%, 59.8% and 35.3%, respectively. This suggests that the application of heat and moisture mobilised free Ca^2+^ and renders it susceptible to chelation and precipitation by phytic acid at elevated pH. The significant changes in the location of Ca^2+^ noted above was likely due to the high phytate regions of the grain attracting and binding free Ca^2+^ likely rendering it unavailable for absorption. It remains to be seen if such changes in diet Ca availability occur during the process of commercial feed manufacture and whether this influences subsequent protein digestion.

## Author contributions

**Edwin T. Moran**: Original Conceptualization, Visualisation, Writing - original draft, Writing - review & editing. **Michael R. Bedford**: Extended conceptualization, Writing - original draft, Writing - review & editing.

## Declaration of competing interest

We declare that we have no financial and personal relationships with other people or organizations that can inappropriately influence our work, and there is no professional or other personal interest of any nature or kind in any product, service and/or company that could be construed as influencing the content of this paper.
